# Distribution of HPV Genotypes and Involvement of Risk Factors in Cervical Lesions and Invasive Cervical Cancer: A Study in an Indian Population

**Published:** 2014

**Authors:** Shikha Srivastava, U P Shahi, Arti Dibya, Sadhana Gupta, Jagat K Roy

**Affiliations:** 1 *Cytogenetics Laboratory, Department of Zoology, Banaras Hindu University, Varanasi 221005, India.*; 2 *Department of Radiotherapy and Radiation Medicine, Institute of Medical Sciences, Banaras Hindu University, Varanasi 221005, India.*; 3 *Indian Railways Cancer Institute and Research Centre, Varanasi, India.*; 4 *Department of Obstetrics and Gynaecology, Institute of Medical Sciences, Banaras Hindu University, Varanasi 221005, India.*

**Keywords:** Cervical cancer, clinical- pathological parameters, genotypes, human papillomavirus, socio-demographic factors, rural area

## Abstract

Human papilloma virus (HPV) is considered as the main sexually transmitted etiological agent for the cause and progression of preneoplastic cervical lesions to cervical cancer. This study is discussing the prevalence of HPV and its genotypes in cervical lesions and invasive cervical cancer tissues and their association with various risk factors in women from Varanasi and its adjoining areas in India. A total of 122 cervical biopsy samples were collected from SS Hospital and Indian Railways Cancer Institute and Research Centre, Varanasi and were screened for HPV infection by PCR using primers from L1 consensus region of the viral genome. HPV positive samples were genotyped by type-specific PCR and sequencing. The association of different risk factors with HPV infection in various grades of cervical lesion was evaluated by chi-square test. A total of 10 different HPV genotypes were observed in women with cervicitis, CIN, invasive squamous cell cervical carcinoma and adenocarcinoma. Increased frequency of HPV infection with increasing lesion grade (p=0.002) was observed. HPV16 being the predominant type was found significantly associated with severity of the disease (p=0.03). Various socio- demographic factors other than HPV including high parity (p<0.0001), rural residential area (p<0.0001), elder age (p<0.0001), low socio-economic status (p<0.0001) and women in postmenopausal group (p<0.0001) were also observed to be associated with cervical cancer.These findings show HPV as a direct cause of cervical cancer suggesting urgent need of screening programs and HPV vaccination in women with low socio-economic status and those residing in rural areas.

Cervical cancer is one of the common female malignancies, ranking first in developing countries and human papillomavirus (HPV) was recognized as the main sexually transmitted etiological agent for the disease. Since 1981 when a relationship was established between papillomavirus and cervical neoplasia (1), more than 100 different genotypes of human papillomaviruses (HPVs) have been recognized. Approximately 40 HPV genotypes were found to be associated with anogenital infections and are generally classified according to their oncogenic potential into low, high and intermediate risk types. HPV16, 18, 31, 33, 35, 39, 45, 51, 52, 56, 58, 59, 68, 73 and 82 were considered as high risk types or oncogenic types due to their presence in high grade squamous intraepithelial lesions (HSIL) or cervical cancer, HPV6, 11, 40, 42, 43, 44, 54, 61, 70, 72 and 81 are considered as low risk types since they are usually associated with benign warts while HPV26, 53 and 66 as intermediate risk types found less frequently in cancer but associated with SILs (2). Presence of HPV has been implicated in 99.7% of the squamous cell cervical carcinoma worldwide (3). It was observed that low risk type HPV6 and 11 were associated with benign lesions (4) while high risk types HPV16 and HPV18 have been linked to squamous cell carcinomas and adenocarcinomas, respectively (5, 6), however, their prevalence in invasive cervical carcinoma and cervical intraepithelial neoplasia (CIN) shows a wide geographical variation.

India is a developing country with 17.2% of the world’s total population (i.e., 1/6th) (7). It has a cervical cancer incidence of 23.5 per 100,000 women which is highest among all the female malignancies (8). In India, approximately 134,420 women were diagnosed with cancer of cervix in the year 2010 and 72,825 died (8). HPV being the most important risk factor, needs to be evaluated in all the regions of the country since the prevalence of HPV and its genotypes are different in the populations from different geographical regions. Its assessment at various stages of the disease will also be helpful in understanding its role in cervical cancer pathogenesis. However, the incidence of cervical cancer and CIN were found to be low relative to high frequency of HPV infection showing that not all the HPV infected women develop cervical lesion and not all the pre-neoplastic lesion get converted into invasive cervical cancer (9). This shows that most of the HPV infections are transient and viral persistence is required for the progression of the disease (9). Therefore, it is important to identify the different factors contributing in viral persistence and disease progression.

This study aimed to determine the prevalence of HPV and its genotypes in different histological grades of cervical specimens of women from Varanasi and adjoining regions. An attempt was also made to find out a correlation of HPV infection with socio-demographic factors forming a definite and effective link with the persistence of virus and disease progression.

## Materials and Methods

**Patients and data collection**

A total of 122 fresh cervical tissue biopsies were collected from Sir Sunderlal Hospital and Indian Railways Cancer Institute and Research Centre, Varanasi, between 2008 and 2010. The age range of the patients was 25-80 years (Mean age=53.3). A questionnaire form along with consent was filled by each female participant. Information regarding marital status, age of conception, parity, smoking, menopausal status, diet, place of residence, socio-economic status, history of HPV infection or any kind of disease was recorded. Based on histopathological reports, all the cases were categorized into cervicitis (inflammatory cervix), mild to moderate dysplasia (CIN1/2/3) and cervical cancer (SCC/ADC). Staging of cervical cancer cases was done according to the International Federation of Gynecology and Obstetrics (FIGO) classification. The control subjects included were randomly chosen healthy normal women without any previous history of HPV infection, hysterectomy, cervical neoplasia/ cervical cancer or any other anogenital cancer. Ethical clearance for the present study was taken from the Institutional Ethics Committee.

**Sample collection and DNA isolation**

Cervical biopsies were collected in vials kept on ice, immediately transported and stored at -80^o^C for further use. In control subjects, exfoliated cells were collected from ectocervix region in 5ml of pre-chilled PBS. DNA was extracted according to the protocol described previously (10) with few modifications. Briefly, tissue was minced, resuspended in 600µl of lysis buffer (0.3% SDS, 1xTE) and incubated with 100µg of proteinase K at 55^o^C for 16 h followed by extractions with phenol:chloroform:isoamyl alcohol (25:24:1 v/v), chloroform:isoamyl alcohol (24:1 v/v). DNA was precipitated with 1/10 volume of 3M sodium acetate and 0.7 volume of isopropanol, dried and re-suspended in TE (Tris- EDTA, pH 8.0) buffer. Purified DNA samples were electrophoresed on 1% agarose gel to check the quality of DNA and its quantity was determined by Nanodrop spectrophotometer (Nanodrop, ND- 1000).

**Detection of HPV DNA**

**Fig. 1:**
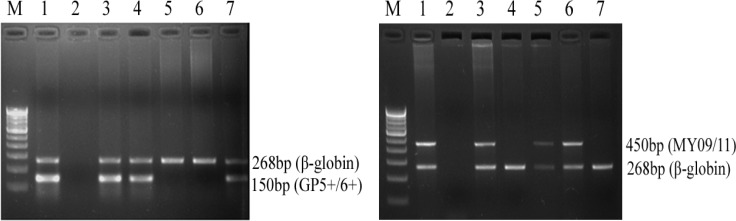
Representative images of agarose gel showing HPV positive cases. Amplification of **(a)** GP5+/6+ primer product along with β-globin as an internal control in genomic DNA samples of cervical cancer biopsies. M: 100bp DNA ladder, lane 1 and 2: HPV positive and negative controls, respectively, lane 3, 4 and 7: HPV positive samples, lane 5 and 6: HPV negative samples. **(b)** MY09/11 primer product along with β-globin as an internal control in genomic DNA of cervical cancer biopsy samples. M: 100bp DNA ladder, lane 1 and 2: HPV positive and negative controls, respectively, lane 3, 5 and 6: HPV positive samples, lane 4 and 7: HPV negative samples

All the samples were first subjected to β-globin amplification (268bp) to determine the quality of DNA by using PC04/GH20 (11) primer set and the samples positive for β-globin were further proceeded for the detection of HPV infection. HPV DNA was detected by PCR using the primers from L1 consensus region of the viral genome. For this, primer sets MY09/11 (12) and GP5+/6+ (13) were used since together they detect the broad spectrum of HPV types at subpicogram level. PCRs were performed in a final volume of 25µl consisting of 100ng DNA samples, 1X PCR buffer (10mM Tris Cl pH 8.3, 50mM KCl, 1.5mM MgCl_2_), 10pmole of each forward and reverse primers, 4µl of dNTP mix (containing 200µM each of dATP, dTTP, dCTP and dGTP), and 0.3U of Taq polymerase (Bangalore Genie). All the sets of PCR reactions were performed along with positive and negative controls. The known HPV positive DNA sample served as a positive control while reaction without DNA served as a negative control. Amplification was performed on thermocycler (Applied Biosystems) and the amplicons were checked on 2% agarose gel giving a product size of 450bp (MY09/11) and 150bp (GP5+/6+) (Fig. 1a, b). The sequences of different primer sets were mentioned in Table 1.

**Table 1:** Primers for detection of HPV and its different genotypes

S. No.	Primer Sequence (5’-3’)	Region	Product Size (bp)	References
MY09/11	FP- GCM CAG GGW CAT AAY AAT GG RP- CGT CCM ARR GGA WAC TGA TC	L1	450	12
GP5+/6+	FP-TTT GTT ACT GTG GTA GAT ACT AC RP-GAA AAA TAA ACT GTA AAT CAT ATT C	L1	150	13
HPV 16	FP- AAG GCC AAC TAA ATG TCA C RP- CTG CTT TTA TAC TAA CCG G	LCR	217	14
HPV 18	FP- TGA GGT ACC ATT CGA TAT TT RP- TAG CAA AAA GCT GCT TCA CGC	L1	118	-
HPV 31	FP- TAA GCT CGG CAT TGG AAA TAC CCT RP- CCT TCC TCC TAT GTT GTG GAA TCG	E6	350	15
HPV 33	FP- AAC GCC ATG AGA GGA CAC AAG RP- ACA CAT AAA CGA ACT GTG GTG	E7	211	16
HPV 35	FP- CCCGAGGCAACTGACCTATA RP- GGGGCACACTATTCCAAATG	E7	230	-do-
β-globin	FP- GAA GAG CCA AGG ACA GGT AC RP- CAA CTT CAT CCA CGT TAC ACC		268	11

Key to symbols: M=A/C; R=A/G; W=A/T; Y=C/T

**Fig. 2:**
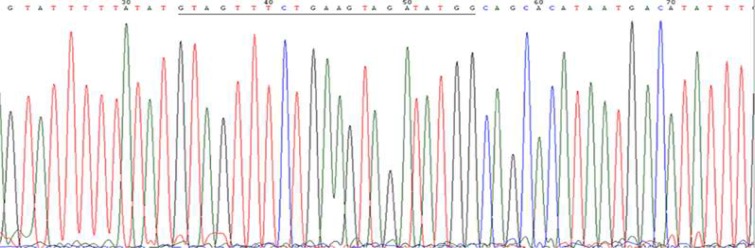
A DNA sequence excised from an electropherogram. Electropherogram showing signature sequence (24 bases underlined) for HPV16 genotype present 34 bases upstream of GP5+ primer binding site

**HPV genotyping**

The samples positive for HPV infection were proceeded for genotyping using type- specific primer sets for high risk HPV types 16, 18, 31, 33 and 35 (14-16). Clones for HPV31, 33, 35 and known HPV16 and 18 positive DNA samples served as a positive control during PCR. Samples negative for the above-mentioned HPV types were sequenced (Fig. 2) and analyzed as described previously (17).

**Statistical analysis**

Univariate association of socio-demographic factors and clinical parameters with the presence and absence of HPV/HPV16 infection in different cervical histology group was estimated by standard contingency table method using Fisher’s exact or Chi-square tests. Two sided p-value was calculated and considered significant when p≤0.05. All the statistical analysis was done by SPSS statistical software (version 16.0).

## Results

Out of 122 biopsy samples obtained, 10 samples were of cervicitis, 15 CINs, 92 squamous cell carcinomas (SCC) and 5 with adenocarcinoma (ADC). All the samples were positive for β-globin and suitable for further genotyping.

Altogether 10 different HPV genotypes were observed, i.e., HPV6, 11, 16, 18, 31, 33, 35, 45, 58 and 86, all of them were high risk types except HPV6 and 11 (low risk types present in cervicitis and ADC group) and HPV86 (intermediate risk type present in ADC group) (Table 2). Among single HPV infection (n=69), HPV16 was the most prevalent type observed in CIN and SCC groups (20% and 42.4%, respectively) while HPV18 (40%) and HPV31 (20%) were the most frequent types detected in adenocarcinoma and cervicitis, respectively. 40 patients were infected with multiple HPV types comprising of two or three genotypes together. In multiple infections, 33 out of 40 cases were positive for double infections, 16/18 (n=15, 37.5%) followed by 16/31 (n=10, 25%). High risk HPV types were detected in all the cases of multiple infections except one showing unknown/low risk types 86/11 in ADC. Triple HPV infections were present in 7 samples of SCC only. HPV16 was present in 71.7% (n=66) of the cases of SCC including single, double and triple infections followed by HPV18 (n=23, 25%) and HPV31 (n=14, 15.2%). In control group, HPV16 was the most frequent type (n=6, 5.7%) observed followed by HPV18, 6, 81 and 31 (see Table 2).

**Table 2:** Distribution of various HPV types in different cervical histology

HPV Types	Cervicitis n=10 (%)	bCINs n=15 (%)	cSCC n=92 (%)	dADC n=5 (%)	Control n=104 (%)	Total
HPV negative	3 (30)	4 (26.6)	4 (4.3)	0	91 (87.5)	102
HPV positive	7 (70)	11 (73.3)	88 (95.6)	5 (100)	13 (12.5)	124
Single Infection 6 16 18 31 33 35 58 81	4 (40) 1 (10) 1 (10) 0 2 (20) 0 0 0 0	4 (26.6) 0 3 (20) 1 (6.6) 0 0 0 0 0	58 (63.0) 0 39 (42.4) 8 (8.6) 5 (5.4) 2 (2.2) 2 (2.2) 2 (2.2) 0	3 (60) 0 1 (20) 2 (40) 0 0 0 0 0	13 (12.5) 2 (1.9) 6 (5.7) 2 (1.9) 1 (0.9) 0 0 0 2 (19)	82
Multiple Infections 16/18 16/31 16/33 16/35 18/35 31/33 86/11 16/18/33 16/18/35 16/31/33 16/31/35 18/31/45	3 (30) 1 (10) 1 (10) 0 0 1 (10) 0 0 0 0 0 0 0	7 (46.6) 3 (20) 3 (20) 0 0 0 1 (6.6) 0 0 0 0 0 0	28 (30.4) 10 (10.8) 6 (6.5) 4 (4.3) 1 (1.1) 0 0 0 2 (2.2) 2 (2.2) 1 (1.1) 1 (1.1) 1 (1.1)	2 (40) 1 (20) 0 0 0 0 0 1 (20) 0 0 0 0 0	0	40
aUnidentified	0	0	2	0		2

a Unidentified Due to inadequate DNA sample;

b CIN: Cervical intraepithelial neoplasia;

c SCC: Squamous cell carcinoma of the cervix;

d ADC: Adenocarcinoma of cervix.

The prevalence of HPV16 infection showed an increasing trend from 30% in cervicitis to 71.7% in SCC (p=0.03). 11 samples (3 cervicitis, 4 CINs and 4 SCC cases) were negative for HPV infection and 2 remain unidentified due to inadequacy of the DNA amount (Table 2).

The prevalence of single and multiple HPV type infection in cervicitis, CIN, squamous cell carcinoma and adenocarcinoma was shown in (Table 2). Single HPV type infection was more frequent in squamous cell carcinoma cases (63%) than CIN (26.6%) while multiple HPV infections were more frequent in CIN (46.6%) in comparison to SCC patients (30.4%) showing a decreasing trend of the prevalence of multiple HPV types with the severity of cervical lesion.

Table 3 shows HPV prevalence in different histologic differentiation stages of cervical tissues. It was observed to be 70% in cervicitis, 73.3% in CIN, 95.6% in SCC and 100% in ADC. It was noteworthy that HPV infection was showing a statistically significant association (p=0.002) with increasing lesion grade. When squamous cell carcinoma samples were categorized (FIGO classification), an increase in the frequency of single HPV infection with the increase in the stage of the disease was observed, i.e., the prevalence of single HPV infection was increased from stage I (50%) to stage III/IV (70%), while the reduced prevalence of multiple type HPV infections were observed (50% in stage I to 20% in stage III/IV). The high prevalence of HPV was observed in well differentiated tumours (96.9%) which were decreased with the decrease in differentiation grade. Although the association of HPV prevalence with FIGO stage of SCC (p=0.298) and histological differentiation grade (p=0.554) was not significant statistically.

Table 4 represents the association of HPV in different cervical histology groups and control samples. We found HPV as one of the most strongly associated factors in all the three histologic groups. Out of the three groups, the risk associated with HPV infection was considerably high for cervical cancer group as compared to other groups, i.e., the women with HPV infection were at 162.7 times greater risk for cervical cancer than those without HPV infection (CI=51.1-517.9, p<0.0001).

Table 5 represents the association of socio-demographic factors other than HPV in different cervical histology groups and control samples. SCC and ADC samples were taken together as cervical cancer cases (due to the small number of ADC samples, n=5). Mean age was observed to be 50.1 years in cervicitis group, 52.5 years in CIN and 53.7 years in cervical cancer while it was 41.8 years in control group showing an increase in the mean age with increasing lesion grade.

Chi-square test and odds ratio showed increasing age (women ≥45 years) as a significantly related factor with cervical cancer (OR= 9.4, p<0.0001) while it was unrelated to the risk of cervicitis and CINs (preinvasive lesions). Further more, the women with high parity (>3) had 3.5 times greater risk for cervical cancer (CI= 1.9-6.4, p<0.0001) while it did not show any association with other histologic groups. Investigation revealed that the women in low socio-economic status was significantly associated with the risk of cervical cancer (OR=3.3, p<0.0001) while those residing in rural areas were associated with the risk of CIN (OR=6.7, p=0.01) and cervical cancer (OR=3.46, p<0.0001) but not with cervicitis. In addition, women in postmenopausal group were at greater risk of developing CINs (p=0.02) and cervical cancer (p<0.0001). Diet, age at first intercourse and religion did not exhibit any relationship with all the three histologic groups.

HPV16 was observed to be the predominant type in all the groups. When samples were categorized according to high risk HPV16 type and other HPV types (excluding HPV16) along with socio-demographic factors (Table 6), a significant association (p=0.02) with place of residence (rural *vs* urban) in CIN category was found. Women living in rural areas and infected with high risk HPV16 were at increased risk of developing CIN and subsequently, cervical cancer (p=0.07). No other factors showed significant association with HPV 16 infection.

## Discussion

Different epidemiological studies reported HPV as one of the important factors constantly associated with cervical cancer, though alone it was insufficient for the progression of cervical pre-neoplastic lesion to invasive cervical cancer. Although not all the HPV genotypes were equally responsible for the development of pre-neoplastic lesion, some of them present in the anogenital regions belonging to oncogenic category were found constantly associated with the disease.

In the present study, the prevalence of HPV and its genotypes was evaluated in the samples of cervicitis (inflammatory cervix), pre-neoplastic lesions (CIN) and cervical cancer. A significant increase in HPV prevalence with increase in severity of the disease (p=0.002) was observed showing HPV as an important risk factor in disease progression (Table 3).

**Table 3:** Association of clinical-pathological parameters with HPV infection

Clinical-pathological parameters	Total Sample No.	Total HPV +ve (%)	Single Infection No. (%)	Multiple Infection No. (%)	Total HPV16 +ve (%)	Total HPV -ve (%)	p-value
Histological Type							0.002*
Cervicitis CIN^b^1/2/3 Squamous Cell Carcinoma (SCC^c^) Adenocarcinoma	10 15 92 5	7 (70) 11 (73.3) 88 (95.6) 5 (100)	4 (40.0) 4 (26.6) 58 (63.0) 3 (60.0)	3 (30.0) 7 (46.6) 28 (30.4) 2 (40.0)	3 (30) 9 (60) 66 (71.7) 2 (40)	3 (30.0) 4 (26.6) 4 (4.3) 0 (0.0)	
FIGO Stage^a^ (SCC)							0.298
I II III/IV	10 38 30	10 (100) 36 (94.7) 27 (90.0)	5 (50.0) 22 (57.8) 21 (70.0)	5 (50.0) 14 (36.8) 6 (20)	8 (80) 28 (73.6) 20 (66.6)	0 (0.0) 1 (1.1) 3 (3.2)	
Histological Differentiation							0.554
Well Moderate Poor	33 32 8	32 (96.9) 30 (93.7) 7 (87.5)	22 (66.6) 17 (53.1) 5 (62.5)	9 (27.3) 13 (40.6) 2 (25)	22 (66.6) 23 (71.8) 6 (75.0)	1 (3.0) 2 (6.2) 1 (12.5)	

* Significant at p value ≤ 0.05

a FIGO stage was not known for all the samples.

b CIN: Cervical intraepithelial neoplasia

c SCC: Squamous cell carcinoma of the cervix

**Table 4:** Risk of cervicitis, CIN and cervical cancer in relation to the presence of HPV infection

Socio-demographic Factors	No. of Control Samples	Cervicitis		CIN		Cervical Cancer	
		No. of cases	Odds Ratio (95% CI)	No. of cases	Odds Ratio (95% CI)	No. of cases	Odds Ratio (95% CI)
HPV							
Negative Positive	91 13	3 7	1.0 (reference) 16.3 (3.7-71.2)	4 11	1.0 (reference) 19.2 (5.3-69.4)	4 93	1.0 (reference) 162.7 (51.1-517.9)

**Table 5:** Association of socio-demographic factors other than HPV in various histologic groups

Socio-demographic Factors	No. of Control Samples	Cervicitis			CIN			Cervical Cancer		
		No. of cases	Odds Ratio (95% CI)	p-value	No. of cases	Odds Ratio (95% CI)	p-value	No. of cases	Odds Ratio (95% CI)	p-value
Age (years)										
< 45 ≥ 45	54 50	2 8	1.0 (reference) 13.1 (1.1-156.8)	0.09	4 11	1.0 (reference) 2.9 (0.88-9.9)	0.09	10 87	1.0 (reference) 9.4 (4.3-20.0)	<0.0001
Parity										
0-3 >3	58 46	4 6	1.0 (reference) 1.8 (0.5-7.1)	0.5	6 9	1.0 (reference) 1.8 (0.62-5.7)	0.2	24 67	1.0 (reference) 3.5 (1.9-6.4)	<0.0001
Age at first intercourse										
≤ 20 years > 20 years	46 58	2 4	0.63 (0.11-3.5) 1.0 (reference)	0.69	5 9	0.70 (0.22-2.2) 1.0 (reference)	0.58	37 41	1.13 (0.63-2.0) 1.0 (reference)	0.76
Socio Economic status										
Middle Low	56 48	8 2	1.0 (reference) 0.29 (0.05-1.4)	0.19	7 8	1.0 (reference) 1.33 (0.45-3.9)	0.78	24 68	1.0 (reference) 3.3 (1.8-6.0)	<0.0001
Diet										
Veg Non-veg	54 50	5 5	1.0 (reference) 1.08 (0.29-3.9)	1.0	10 5	1.0 (reference) 0.54 (0.17-1.7)	0.41	59 33	1.0 (reference) 0.60 (0.34-1.0)	0.11
Place of residence										
Urban Rural	53 51	7 3	1.0 (reference) 0.44 (0.10-1.8)	0.3	2 13	1.0 (reference) 6.7 (1.45-31.4)	0.01	21 70	1.0 (reference) 3.46 (1.86-6.44)	<0.0001
Menopause status										
Post Pre	35 69	6 4	2.9 (0.78-11.1) 1.0 (reference)	0.16	10 5	3.9 (1.2-12.4) 1.0 (reference)	0.02	79 13	11.9 (5.8-24.4) 1.0 (reference)	<0.0001
Religion										
Hindu Muslim	96 8	9 1	1.0 (reference) 1.3 (0.14-11.8)	0.5	15 0	1.0 (reference) 0.36 (0.02-6.6)	0.5	91 6	1.0 (reference) 0.79 (0.26-2.37)	0.78

**Table 6:** Socio demographic factors in relation to HPV16 vs other HPV types in different cervical histologic groups

Socio-demographic Factors	Cervicitis				CIN^b^				Cervical Cancer			
	HPV16	Others^a^	χ^2^	p-value	HPV16	Others^a^	χ^2^	p-value	HPV16	Others^a^	χ^2^	p-value
Age (years)												
≤ 50 > 50	3 0	4 1	0.87	0.35	3 6	1 1	0.19	0.65	27 38	14 9	2.55	0.11
Parity												
0-3 >3	0 3	2 2	2.10	0.14	4 5	1 1	0.02	0.88	16 48	7 16	0.25	0.61
Age at sexual intercourse												
≤ 20 years > 20 years	0 2	2 0	1.00	0.31	2 7	1 1	0.64	0.42	27 27	9 12	0.31	0.57
Socio Economic status												
Low Middle	1 2	1 3	0.05	0.81	6 3	1 1	0.19	0.65	50 15	17 6	0.08	0.77
Diet												
Veg Non-veg	2 1	2 2	0.19	0.65	6 3	2 0	0.92	0.34	44 21	13 10	0.93	0.33
Place of residence												
Rural Urban	0 3	2 2	2.10	0.14	9 0	1 1	4.95	0.02*	47 17	21 2	3.16	0.07
Menopause status												
Pre Post	1 2	2 2	0.19	0.65	2 7	1 1	0.63	0.42	9 56	4 19	0.17	0.68

* Significant at p value ≤ 0.05,

a All HPV types excluding HPV16,

b CIN: Cervical intraepithelial neoplasia

The high prevalence of HPV infection was observed in squamous cell carcinoma patients (95.8%) which was consistent with the previous reports (90.3% in Italy (18), 98.7% in Venezuela (19) and 97% in Paraguay (20)). 70% of HPV infection was observed in cervicitis and 100% in ADC which was high compared to the available reports in different populations (14.2% in cervicitis cases as observed in China, 37.5% in Ecuadorian women (21, 22) and 94.1% in adenocarcinoma in Italy (18)) probably due to small sample size, 10 in case of cervicitis and 5 in ADC.

Single HPV infection was seen more frequently in SCC (63%) than CIN (26.6%) in contrast to multiple HPV infections which were observed more commonly in CIN (46.6%) than SCC (30.4%). Such observations were in correlation with previous studies (10, 23). FIGO stages of SCC were also showing an increase in the percentage of single HPV type from stage I (50%) to III/IV (70%) while there was a decrease in the frequency of double and triple HPV infection with increasing clinical stage implicating the prevalence of single genotype with the increasing clinical stage of the disease. However, no such correlation was observed in the study by Munagala *et al*. (24) with stage, grade or histologic type of the disease.

The entire single as well as multiple HPV infections reported in our study in all the cases of CIN and SCC was of high risk types. Hildesheim *et al*. (9) reported that high risk HPV types persist longer than low risk types by resisting host immunity. In case of co-infections with different high risk HPV types, only few are capable of segregating and able to persist in the later stage of the disease (10). This segregation occurs at the stage of CINIII and cervical carcinoma leading to the emergence of a single dominant HPV type, thus, converting heterogeneous HPV population to homogeneous one (23, 10). Matsukura and Sugase (25) reported that different HPVs have different potencies for inducing various grades of CIN depending upon the similarities in their nucleotide sequence with HPV16 viral genome. They categorized HPV16, 31, 33, 35, 52, 58 and 57 in one group due to their capability of inducing high grade dysplasia or CINIII.

We identified HPV16 as the most prevalent type observed in all the cervical histological groups showing significant increase (p=0.03) in frequency with the increase in severity of the lesion, being highest in SCC (71.7%) and lowest in cervicitis (30%) thus, supporting its importance in the pathogenesis of cervical cancer. The high incidence of HPV16 in squamous cell cervical carcinoma was comparable to the prevalence reported worldwide, i.e., 65.2% in Saudi Arabia (2) and 65.1% in Shandong province, China (21). In India, also the difference in the prevalence of HPV16 was reported in different regions of the country being 66.7% in Andhra Pradesh (26), 74.3% North India (27), 81.7% Sevagram (28), 63% South India and 50% in the eastern Indian population (29). A study including different geographical regions of India also reported the highest prevalence of HPV16 in Chennai (88%) while the lowest in Jammu and Kashmir (14.2%) which may be due to circumcision in Muslim population (30).

In our study, HPV18 was the next most frequent genotype detected after HPV16 and both of them comprise of 79% of the total HPV infection in SCC. Earlier findings also suggested that high grade SIL infected with HPV16, 18 or 45 have more probability of progression to SCC which may be due to either of their greater potential to transform non-malignant lesion to malignant one or due to their ability to evade host immune system (31).

We found HPV as the most strongly associated factor in all the histologic groups (see Table 4) which was in parallel with the available literature (32, 33).

Another aim of our study was to investigate the cofactors in all three histologic groups other than HPV (shown in table 5). We observed the women in older age group (>45 years) were at greater risk of developing cervical cancer which was in parallel to the previous report (34) while no correlation was observed with cervicitis and CIN categories.

We also found high parity (>3) as one of the important risk factors in this population. This may be due to early marriages in India which resulted in higher number of pregnancies. It was reported that high parity leads to four times increased risk of squamous cell cervical carcinoma (35).

We also observed rural residential area as a risk determinant of preinvasive and invasive cervical carcinoma. This has been supported by several studies in India (36, 37) as well as other regions of the world (32).

Low socio-economic status was also one of the significant factors found to be associated with cervical cancer in our study population which was in parallel to the other studies (38, 39). Similar observations were also reported in cervical cancer cases from northern Nigeria where low socio-economic level was one of the risk factors in the development of cervical cancer (40). The high incidence of cervical cancer has been reported in rural and low income group (41) which may be due to poor health, poor genital hygiene in females and their partners, low education level, lack of awareness among people, lack of organized screening programs, their inaccessibility to health care services and social stigma which prevent the affected individuals from seeking medical attention. Effective prevention and early diagnosis of cervical cancer and HPV infection is needed to reduce the cervical cancer cases.

Women in postmenopausal group were at increased risk of developing cervical cancer as observed in our study which was also consistent with the findings in other studies (42) thus suggesting the need of routine HPV screening and pap smear tests in older age groups also. Other cofactors including diet, age at first intercourse and religion did not show any association.

HPV16 was the predominant type observed in all the grades of cervical lesion and women infected with HPV16 and living in rural areas were at increased risk of developing CIN (p=0.02) which may later develop into cervical cancer (p=0.07) (Table 6). In our study with asymptomatic women population of the same region (Varanasi and adjoining areas), high prevalence of HPV16 was observed showing a significant association of HPV16 infection in rural population (17). This shows that HPV infection, HPV16 being the predominant one, is one of the important risk factors in the progression of cervical lesion for women living in rural areas under study. Different regions of India also showed the high prevalence of HPV infection in cervical carcinoma patients residing in rural areas. A study conducted in rural areas of Medchal Mandal of Andhra Pradesh and Sevagram of Central India also showed 87% and 93% HPV positivity, respectively in invasive squamous cell carcinoma (26, 28).

Our findings suggested the urgent need of HPV and cervical cancer screening programs in women belonging to low socio-economic group and those residing in rural areas to detect abnormal/cancerous cells at initial stage of the development and to provide treatment which could be better responded at early stage of the cancer. Since India alone bear a burden of 1/5 of total cervical cancer cases all over the world and almost 70% of the population in India resides in villages so this study becomes more relevant. Vaccine targeting HPV31, 33, 35 and 58 apart from HPV16 and 18 (covered under Gardasil and Cervarix vaccines) could help prevent the large number of cervical cancer cases. Increasing frequency of HPV/HPV16 infection with increasing stage of the disease shows a direct link of HPV with the disease severity. Since these findings relied on small number of cases, further studies with large number of cases may be of significance to support these findings.
